# Investigating Factors Responsible for Teacher Burnout in English as Foreign Language Classes

**DOI:** 10.3389/fpsyg.2022.876203

**Published:** 2022-05-31

**Authors:** Yingli Cheng

**Affiliations:** School of Electrical and Information Engineering, Ankang University, Ankang, China

**Keywords:** teachers’ burnout, negative emotions, English as Foreign Language (EFL), language studying, academic achievement

## Abstract

Regarding the considerable role educators have within the area of studying language, their affections and sensations have currently been at the core of attention. Such affections and feelings can both elevate their career achievement or impede their achievement such as burnout. Indeed, they usually experience “burnout” because of their stressful job. As an issue, educator burnout should be considered because it has an influence both on learners and educators. To gain a setting with lower stress and prevent mental and physical burnout in students as well as the educators, the present article investigated the issue of burnout and the affective factors. This mini review aims to provide the definition, background, and the causes of burnout along with some implications for language stakeholders are presented.

## Introduction

One of the most anxiety-provoking careers is becoming an educator (Gu and Day, 2007) while they are considered as important backbones in driving the educational and instructing cycle in any academic environment and have an important effect on learners’ presentation as well as school standards (Scheopner, 2010). Within previous years, educators have become the center of consideration in academic institutions as they have the most significant function in instructional settings (Pishghadam et al., 2021). However, for the last 40 years, around the world, specifically, instructing is an anxiety-provoking profession, and educator anxiety and burnout are global occurrences (Stoeber and Rennert, 2008; Liu and Onwuegbuzie, 2012). Indeed, education is a career with a rather high probability of burnout since prolonged anxiety can result in chronic fatigue, which is closely associated with burnout (Ryu and Kim, 2020). In numerous nations, educators quit the instructing career and seek another job outside school, primarily because of different setting-specific elements like low wages, poor career circumstances, absence of managerial help, high-paying substitutes outside of school, and family-related worries (Liu and Onwuegbuzie, 2012; Skaalvik and Skaalvik, 2017). Indeed, they are referred to the notion of “burnout” that has been the first and foremost concern as a global case (Aloe et al., 2014a) mirroring people’s awareness of unsatisfied demands and expectations, which result in a three-way condition of fatigue, depersonalization, and reduced individual achievement associated with increasing disappointment and deconstructive connotations of undermining, exhaustion, unconcern, sadness, and low confidence (Steinhardt et al., 2011). Burnout is characterized as a decrease in involvement in the sense that a significant, purposeful, and demanding career becomes a displeasing, unsatisfying, and pointless one; therefore, burnout occurs when fatigue is substituted by a sense of energy; skepticism by hope and engagement; and inefficiency by a sense of effectiveness (Maslach et al., 2001). As stated by Maslach (2015), burnout is a mental condition that is brought about by chronic emotive and relational anxiety at work. Thus, anxiety must be eliminated to reduce the degree of burnout. Foreign language educators, especially English educators, are more prone to suffer from educator burnout because of the difficult requirements at work and great degrees of anxiety that are associated with aiding learners in attaining achievement in their language education (Meidani et al., 2019). Furthermore, educators’ role can’t be neglected in second language training/studying because their burnout negatively affects learners’ learning attainment (Roohani and Dayeri, 2019).

In the review of literature, a bulk of research has been done concerning teacher burnout and its elements such as work milieu and personality factors in EFL circumstances (Goddard et al., 2006; Skodova and Lajciakova, 2013; Goetz et al., 2015); however, to the best of the researcher’s acquaintance with the issue, there is the dearth of related review among foreign language teacher burnout. Consequently, this review endeavors to bridge this lacuna by detecting the aspects manipulating EFL teacher burnout.

## Review of the Literature

### Burnout

Burnout has three aspects, which are also regularly found in EFL educators’ burnout (Maslach and Leiter, 2016). The first aspect is emotional exhaustion that alludes to a circumstance in which educators lack emotive energy to keep up with a learner’s education and manner because of prolonged encounters with different inconveniences (Arens and Morin, 2016). In addition, emotional exhaustion is a consequence of being overwhelmed and exhausted by being with others, specifically, learners (Fathi et al., 2021). The second aspect is depersonalization, which is a circumstance in which the educator does not show emotions to the learners and regards them as objects as opposed to individuals (Sas et al., 2011). Depersonalization can be a consequence of emotive requirements of the career as an educator that could deny educators the ability to be reactive to learners’ needs (Maslach et al., 2001). Weak personal efficacy is the feeling an individual has of being less beneficial and competent in their work. It refers to a negative assessment of their career performance and the overall value of their career (Leiter et al., 2014). Individual success is the last aspect that demonstrates whether or not educators have a sense of achievement concerning their careers (Skaalvik and Skaalvik, 2017).

#### Sources of Burnout

It is maintained that multiple elements cause educator burnout (Maslach et al., 2001). Personal elements involve demographic or character factors (age, gender, years of instructing experience, character, etc.). Outcomes regarding age as an indicator of educator burnout are rather inconsistent in the literature. For example, some research has no proof of age as an indicator of educator burnout (Zabel and Zabel, 2001). In research that discovered gender differences, consistent results involved female educators documenting greater degrees of emotive fatigue and male educators documenting greater degrees of depersonalization and ineffectiveness. As for marital status, single people (men in specific) are more likely to have burnout than married people. Unmarried individuals appear to have even greater degrees of burnout than divorced individuals (Maslach and Leiter, 2016). Concerning ethnicity, very little research has evaluated this demographic factor; thus, it is impossible to outline any experiential patterns (Maslach et al., 2001). Also, the outcomes demonstrated that the most effective elements in the condition of burnout are the emotive dimensions of character and career inconveniences like career anxiety and societal help (Nagy and Takács, 2017). Among the career inconveniences, the discerned workload had the strongest effect on the aspect of emotive fatigue and affected this burnout element with the greatest regression coefficient. It is demonstrated by this outcome that workload is a deciding element for the growth of the condition. The discerned workload is linked to the degree of obligation a person intends to accept and the discerned function anxiety a person encounters. Therefore, function anxiety and obligation could be intermediary elements between burnout and discerned workload (Nagy and Takács, 2017).

Other reasons for personal burnout in educators determined in the literature are decreased independence and self-effectiveness (Skaalvik and Skaalvik, 2017), (decreased self-esteem is the capability of effectively coping with a particular circumstance); inflexibility regarding criteria, goals, and functions; low expectations of school administration and instructing institutions, specifically if the latter is linked to a school institution that is not open to alteration and innovation (Aloe et al., 2014b). Institutional elements involve organizational and career features like unsuitable career requirements, socioeconomic status of the school, managerial help, etc (Evers et al., 2004). Maslach et al. (2001) have determined six kinds of institutional inconveniences: career overload, absence of authority (dispute circumstances or function uncertainty), inadequate fulfillment, fall of the sense of civic and belonging (when teamwork is deficient, and so, respect is absent), unfair management within one institution, and so on.

Elements that may increase educator burnout involve an absence of societal help from peers and managers (Maslach et al., 2001), lower socioeconomic school status, institutional inflexibility, career requirements overload, insufficient payments or assets, inadequate educator training or coaching in handling issues involving controlling learners, absence of educators’ involvement in school decision making, and other physical factors like crammed classes, poor workplaces, and poor career circumstances (Seifalain and Derakhshan, 2018). Overall, societal help offers chances for reassessments and adjustive reactions to career anxiety leading to alleviated burnout (Kahn et al., 2006). Furthermore, heavy workloads involve offering feedback on tasks and getting ready for exams and assessments. Career pressure leads to burnout in all dimensions. Moreover, the speed of the condition’s growth is increased by career requirements (Goddard et al., 2006). Studying educator burnout in the setting of EFL is an important topic as outcomes of studies demonstrate that educator burnout can harm learners’ education and inspiration. For example, Shen et al. (2015) showed that an important environmental element is educators’ burnout status, which is associated with the standard of learners’ inspiration.

## Concluding Remarks

Burnout is a crucial mental condition that can impact both human wellbeing and different institutions like schools. EFL educator programs must provide personal and group guidance services to educators who are prone to burnout and must manage educators regularly to monitor alterations in their way of living and standard of instructing. Such programs need to aim at enhancing educators’ interpersonal relationship competencies and to increase their coping tactics to allow them to deal with challenges at work and prevent educator burnout (Akbari and Eghtesadi, 2017).

## Implication and Suggestions for Further Research

This review has important pedagogical suggestions both for educators’ teaching and for foreign language learning. Undeniably, the present review has a few applied practices for the university members and educational employees operating in universities and for the higher education policy-makers. Cognition about the root of burnout is essential for educational members so that the reasons for burnout are diagnosed and removed. Such information is vital due to the fact that specific elements are robust and superb predictors of burnout. The organizational staff must be mainly worried regarding the equipment they offer to their fellows. The rules and processes relating to promoting, financing, rewarding, payments, performance assessment, corporation justice, etc., ought to be cautiously developed and implemented. The management of universities needs to hold managing courses regularly so that the educational team improves vital capabilities and competencies to manage burnout at the workplace. Higher education policy-makers have to design tactics for enhancing the quantity of university financial assets. Furthermore, academic policymakers are encouraged to introduce intervention programs that teach educators about the condition and aid with reducing burnout and anxiety. These kinds of programs can be distinguished based on the direction and purpose of the planned treatment. Therefore, addressing the causes of burnout at the personal level include preparing educators for dealing with anxiety-provoking circumstance; guiding educators’ convictions and awareness, building and enhancing educators’ leadership skills, problem-solving, and dispute solution.

As for the institutional level, techniques could involve lowering the level of polarization in the class, lowering the number of students in every classroom, and altering educators’ career arrangements or instructing arrangements. It could also include developing a helpful environment in the school, creating chances for interaction, allowing for participation in decision making, and building a constructive and open institutional setting. Educators and organizations need to distinguish issues that result in burnout and take measures to evade displeasing and demotivating elements. To eliminate mental and physical indications of burnout, both systematic and individual precautions are required.

By motivating more research in this area, pre and in-service teachers will learn to alter their instructing methods and materials to help counteract the deconstructive effects of anxiety and worry and choose more encouraging ones that will aid them with reducing the level of burnout. This review could aid educators with identifying methods to control or evade burnout, criteria to come up with plans that identify and treat burnout issues, and guides who direct educators in evading burnout by handling its triggers. Moreover, it could offer college syllabus planners insight for changes that offer novice educators techniques to evade burnout by conquering its triggers. Burnout can result from an absence of managerial help and direction on class administration, lesson arrangements, and instructing material; thus, personnel like managers are encouraged to assist with educational and class administration problems.

It is significant for organizations to assume accountability for improving the ecological setting to lower the degree of educators’ burnout. When creating policies regarding the well-being of educators, the administration must pay attention to educators’ perspectives and include them in the policy-creating procedure. Since the policy is founded on field research among educators rather than being planned by management, the “bottom-up” technique in creating policies could be better than the “top-down” one. Further experiential studies can be done, specifically in a qualitative way, through intermediary tools to gain more insight into the function of educator burnout in language circumstances. Further experiential and educational examinations are essential to investigate the mediating contributions of some problems to reduce educators’ burnout. Further studies should be conducted on the effect of career improvement and pertaining educator factors to specify their lengthy influence on educator burnout. This study is a mini review, more longitudinal research is recommended to be conducted to discover the role of various factors on the experience of burnout and find their causal connection with burnout in the area of second language education.

## Author Contributions

The author confirms being the sole contributor of this work and has approved it for publication.

## Conflict of Interest

The author declares that the research was conducted in the absence of any commercial or financial relationships that could be construed as a potential conflict of interest.

## Publisher’s Note

All claims expressed in this article are solely those of the authors and do not necessarily represent those of their affiliated organizations, or those of the publisher, the editors and the reviewers. Any product that may be evaluated in this article, or claim that may be made by its manufacturer, is not guaranteed or endorsed by the publisher.
